# The pholcodine Case. Cough Medicines, IgE-Sensitization, and Anaphylaxis: A Devious Connection

**DOI:** 10.1097/WOX.0b013e318261eccc

**Published:** 2012-07-15

**Authors:** E Florvaag, SGO Johansson

**Affiliations:** 1 Laboratory of Clinical Biochemistry and Section for Clinical Allergology, Department of Occupational Medicine, Haukeland University Hospital, Bergen, Norway; 2 Institute of Medicine, University of Bergen, Norway; 3 Clinical Immunology and Allergy Unit, Department of Medicine, Karolinska Institute, Stockholm, Sweden; 4 Department of Clinical Immunology and Transfusion Medicine, Karolinska University Hospital, Stockholm, Sweden

**Keywords:** Over-the-counter cough medicines, IgE antibodies, pholcodine, anaphylaxis

## Abstract

The Scandinavian data on pholcodine (PHO) strongly indicates that there is a biological chain from PHO exposure through IgE-sensitization to IgE-mediated anaphylaxis to neuromuscular blocking agents (NMBA). PHO is probably one of the strongest inducer of an IgE antibody response known. Of individuals taking PHO in cough medicines, over-the-counter accessibility to large populations, as many as 20 to 25% may become IgE sensitized. Once sensitized, PHO re-exposure will booster IgE antibody levels and IgE by around 100-fold. PHO is monovalent for 2 non-cross-reacting epitopes the quaternary ammonium ion (QAI), the main allergenic epitope of NMBA, and a non-QAI epitope. Thus, PHO most unlikely would initiate an allergic inflammatory response. Consequently, IgE sensitization is not revealed by obvious clinical signs, neither through tests based on IgE-sensitized effector cells. Therefore, it will escape detection if not assayed serologically. However, when subjected to general anesthesia, and thus the IgE-sensitized individual is administered a bivalent NMBA intravenously, the unrecognized presence of serum IgE antibodies to QAI may increase the risk of anaphylaxis 200- to 300-fold. Severe damages to patient's health can result, and mortality rates of 3 to 10% are reported. The Scandinavian experience indicates that the chain of events can efficiently be avoided by stopping PHO exposure: Within 1 year, the prevalence of IgE sensitization to PHO and QAI decreases significantly, and after 2 to 3 years, the numbers of reported anaphylactic reactions decreases equally so.

## 

In recent years, increasing evidence for a connection between the consumption of pholcodine (PHO), an opioid antitussive, in cough and cold medicines and IgE-mediated anaphylactic reactions to neuromuscular blocking agents (NMBA) have been presented. The issue is, by nature, primarily a challenge for doctors and researchers within anesthesiology and allergology but not less for regulatory authorities and national marketing authorization holders. Some are concerned, and others obviously less disturbed by the warning signals thus conveyed. The latter position is often promoted by individuals and official bodies obviously not fully taking into account the mechanisms by which PHO seems to work. However, the consequences are to be born by large populations of individuals around the world continuing to consume PHO.

Therefore, it seems appropriate to review published data on this, in many ways exceptional and devious connection.

## Cough Medicines

Cough and cold medicines in a variety of formulations and pharmacological combinations, often easily accessible without prescription [over-the-counter (OTC)], are widely consumed by most populations world wide. Furthermore, they are often repeatedly taken because viral and bacterial infections of the airways are considered close to normal repetitive afflictions appearing in large groups of individuals. They represent a major body of pharmacological exposure to populations, and thus, national regulatory authorities are expected to monitor carefully the effectiveness against the risks of side effects to the very many exposed.

The effectiveness of available OTC drug combinations for cough and cold can be questioned. A Cochrane review including 26 trials in adults and children recently published [1] concluded that "There is no good evidence for or against the effectiveness of OTC medicines in acute cough." In part, this rather careful conclusion was arrived at because of considerable differences in study characteristics and quality.

## Pholcodine

A major antitussive constituent in a number of these remedies is the morphine (MOR) analog PHO. Since the 1960s, PHO has been included as a constituent in an increasing number of this class of medicines and, thus, consumed in different degrees in countries around the world. Following concerns that use of PHO in cough medicines may put people at the risk of developing anaphylactic reactions to NMBAs used during surgery, quite recently, the European Medicines Agency completed a review of the safety and effectiveness of PHO. According to their recent press release,[2] the Committee for Medicinal Products for Human Use of the European Medicines Agency has concluded that the existing evidence of the risk was weak. Therefore, it recommended that, "all marketing authorisations for medicines containing pholcodine should be maintained throughout the European Union (EU)." It further recommended (Table 1) that, "Patients and health care professionals are reminded that the benefits of pholcodine continue to outweigh its risks for the treatment of non-productive cough. No new risks have been identified with pholcodine" and "Patients taking pholcodine- containing medicines can continue to do so, and should contact their doctor or pharmacist if they have any questions about their treatment." However, further studies were asked for.

**Table 1:** Main Conclusions From the Recent Review on the Safety And Effectiveness of PHO by the European Medicines Agency's Committee for Medicinal Products for Human Use [2]

1	The benefits of pholcodine continue to outweigh its risks
2	No new risks have been identified with pholcodine
3	Although a cross-sensitization between pholcodine and NMBA is biologically plausible, the available data are weak and not fully consistent
4	Patients taking pholcodine-containing medicines can continue to do so

**Figure 1:**
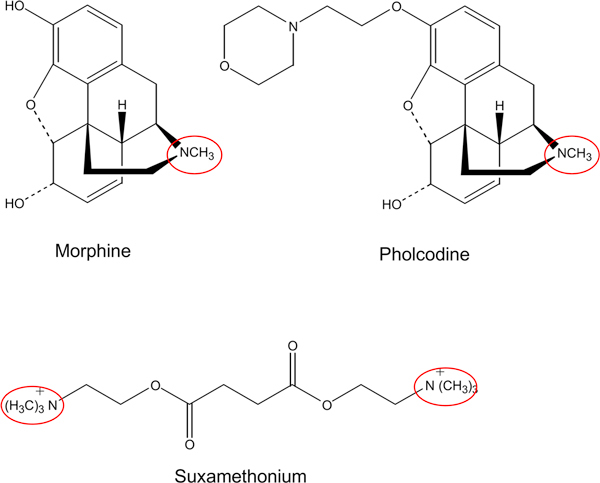
**Schematic representation of morphine, pholcodine, and, the NMBA, suxamethonium**. The IgE-binding epitopes, the quaternary/tertiary ammonium epitopes are encircled.

In principle, PHO (3-(2-morpholinoethyl)morphine), originally synthesized in France in the 1950s, is a MOR molecule with a morpholino side chain (Figure 1). The modification changes the pharmacological mode of action. There is no depression of respiration, pain relief, and CNS excitation, and it is void of euphorizing properties and risk of addiction. PHO is a mild cough suppressant acting directly on the cough center of the CNS, and it has become a widely used cough medicine in numerous formulations. Pharmacokinetically, it is much more slowly eliminated from the body than opioids like codeine, and the concentration in saliva becomes 3 to 4 times higher than in plasma,[3] properties that might add to its immunogenicity.

There are several manufacturers of PHO on the international scene. The international sales are monitored by the United Nations International Narcotics Control Board (INCB) http://www.incb.org. According to their 2008 report, the global production of PHO amounted totally to 6.7 tons where France (3.2 tons), the United Kingdom (1.4 tons), and Hungary (768 kg) ranged as the major PHO-producing countries together accounting for approximately 81% of world production. Total registered export was 3.8 tons with the United Kingdom, Hungary, and Norway as the main exporting countries. Major importing countries were Hong Kong, Pakistan, Australia, and Algeria. Global consumption reached 7 tons equivalent to 140 million sold defined daily doses (S-DDD), and 96% was consumed in the form of preparations listed in Schedule III of the UN 1961 Convention On Narcotic Drugs.

PHO-containing cough medicines are available in many countries around the world and spread over most continents except the United States. The requirements for PHO by the nations of the world also are published by the INCB. In Table 2, we have listed the 29 countries requiring 1 kg or more of PHO in 2012. However, Table 2 indirectly shows that the absolute majority of nations are nonconsumers and, thus, they have decided to meet their inhabitant's demand for antitussive and cold medications without the use of PHO.

**Table 2:** Overview of Official World Requirements for PHO as Given by the United Nations INCB as per January 2012

Continent	Country	2012 Official PHO Requirements (kg)
Africa	Algeria	2500
	Morocco	106
	Nigeria	20
	South Africa	215
	Tunisia	22
	Zimbabwe	3
United States		
Asia	Afghanistan	100
	China	410
	Hong Kong	2000
	Egypt	200
	Malaysia	200
	Nepal	7
	Pakistan	3206
	Singapore	15
Europe	Albania	1
	Belgium	230
	Bosnia and Herzegovina	28
	France	4050
	Ireland	300
	Italy	300
	Macedonia	150
	Netherlands	50
	Poland	47
	Slovenia	9
	Spain	5
	Switzerland	10
	United Kingdom	1000
Oceania	Australia	850
	New Zealand	35
	N = 29	

The requirements are reported in kilograms, and only countries requiring 1 kg or more are listed.

Important for understanding the connection between PHO and NMBAs are the structural relationships (Figure 1). It has for close to 30 years now been known that the major IgEbinding epitope of the NMBA contains the quaternary ammonium ion (QAI) or its tertiary variety [4]. Our studies have shown that PHO, and its parent molecule MOR, contains the QAI epitope, which they share with NMBAs. However, in addition, another epitope of unknown structure on PHO and MOR, not present on NMBAs, was found based on ImmunoCAP (IDD; Thermo Fisher Scientific, Uppsala, Sweden) inhibition studies where IgE antibody binding to QAI, that is, suxamethonium (SUX), is completely inhibited with PHO and MOR, but, the binding to PHO and MOR is only partially inhibited with QAI [5]. Thus, important to note, PHO, functionally monovalent for the 2 non-cross-reacting IgE-binding structural sites, can in principle not initiate an allergic reaction toward itself through cross-binding of IgE antibodies. Therefore, tests dependent on such cross-binding like skin tests, histamine release and basophil activation tests, oral provocation tests, and, not least, intake of cough medicines will come out negative with PHO in individuals who are IgE sensitized to PHO. On the other hand, the NMBAs like SUX and rocuronium are structurally bivalent for the QAI epitope. As such, they appear functionally as allergens that through the cross-binding of IgE antibodies on the surfaces of effector cells can induce immediate allergic inflammatory responses and enhanced by way of administration, eventually leading to anaphylaxis.

The main message of this structure-function relationship is that the basic side effect of PHO, namely, its formidable IgE-sensitizing properties, will go largely unnoticed if only monitored by spontaneous adverse event reporting and not looking specifically for IgE antibodies. Only occasionally will it become clinically manifest like through an anaphylactic reaction to a NMBA, through the referral of patients to allergy clinics for elevated IgE and IgE antibodies of unclear etiology and questionable clinical consequence, or through the odd patient with severe allergic bronchial asthma who is excluded from the treatment option with anti-IgE because the IgE apparently unexplained has become too high and out of range for the capacity of this treatments.

## IgE Sensitization

### Primary Sensitization

**Figure 2:**
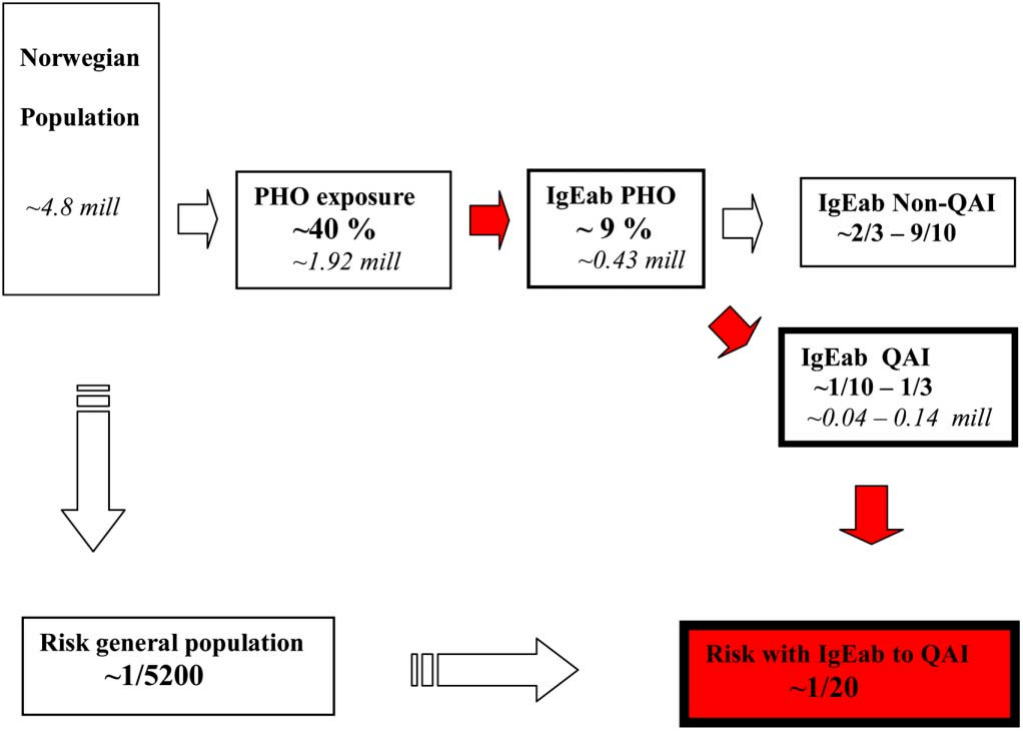
**Relationship between pholcodine exposure, IgE sensitization, and quaternary/tertiary ammonium epitopes (QAI) and estimated risk of IgE-mediated anaphylaxis when receiving suxamethonium during induction of general anesthesia in Norway**. IgEab, is the abbreviation of IgE antibodies. The relationships depicted apply for the years before 2007 when pholcodine (PHO) was taken off the Norwegian market. PHO exposure is given as estimated by the former marketing authorization holder in Norway. Percentage IgE-sensitized to PHO and the relative distribution of IgE specificities to the QAI and the non-QAI epitopes of PHO, respectively, is taken from studies by Florvaag et al. [5,6]. The risk estimates for anaphylaxis during general anesthesia with NMBAs for the general population and individuals IgE sensitized to the QAI epitope before anesthesia with suxamethonium are taken from Harper et al. [22]. Risk is given as the ratio of anaphylactic reactions to number of uneventful general anesthesias using a NMBA. Approximate number of populations in millions is given in italics.

Our present view on the connection among PHO exposure, IgE-sensitization, and anaphylaxis is summarized in Figure 2. There does not exist randomized controlled trials to evidently document the primary IgE-sensitizing potential of PHO, that is, how many of those exposed develop IgE antibodies to PHO. For many years now, such study protocols would probably not pass research ethic evaluations based on present knowledge, at least not in Scandinavia.

However, a considerable body of data supports that primary sensitization by PHO really takes place. Seroepidemiological studies comparing Norway and Sweden, at the time of study representing high-PHO-consuming and non- PHO-consuming countries, respectively, have shown that in Norway, 6% of blood donors and 11% of those with allergy were IgE sensitized to PHO, whereas in Sweden, not one single sensitized individual was found [5].

PHO was withdrawn from the Norwegian market by the marketing authorization holder in March 2007 because of the suspected link between national PHO consumption and IgE sensitization on one hand, and the number of reported cases of NMBA anaphylaxis on the other. However, the national drug authorities were also much concerned about the remarkable effect of PHO on the control mechanisms of IgE production (see below). As a result, the prevalence of sera IgE sensitized to PHO among those with allergy decreased from 11% to 2.7% in 2010, that is, after 3 years without PHO available OTC or by prescription [6] but, still for some time, at the individual's "drug store" at home. Also the number of anaphylaxis decreased significantly. Thus, the decrease in exposure in Norway coincided with a significant decrease in the prevalence of IgE sensitization and reported cases of anaphylaxis. The same had been observed in a retrospective study from Sweden [7].

An international multicenter study comparing consuming and nonconsuming countries, found a significant association (*P *< 0.03) between the national consumptions of PHO, according to the INCB, and the percentages of sera with IgE antibodies to PHO using Phadiatop (IDD; Thermo Fisher Scientific) positive allergics as screening populations [8]. For 2 countries in this study, the Netherlands and the United States, the results were interesting because both showed IgE sensitization to PHO but neither had known PHO-containing drugs on their markets. This could either mean PHO sensitization through unofficial drugs or other means, or mean that other substances than PHO also could induce IgE antibodies. We found it a highly interesting challenge to look for such, probably QAI-containing, substances in the environment. There are indeed many candidates among the daily life chemicals and drugs, but we have after considerable efforts yet not been able to find any with sensitizing properties [9]. However, others argument that this failure discounts the PHO hypothesis [2,10].

The former Norwegian marketing authorization holder estimated that approximately 40% of the population was exposed to PHO through their products. According to the published sensitization data,[5] this could indicate that as many as 1 in 4 to 5 of exposed individuals may develop IgE antibodies to PHO.

At the time when PHO was on the Norwegian market, one observed that those with allergy with IgE levels between 1000 and 5000 kU/L and more than 5000 kU/L were in approximately 30% and 75%, respectively, associated with IgE-sensitization to PHO [6]. Consequently, a major part of patients presenting highly elevated IgE could be correctly diagnosed, and, better still, treated by seeing to that PHO exposure ceased and thereafter monitoring levels toward normalization. Both reported data and unpublished observations showed that in most individuals, IgE antibodies decreased to less than 0.35 kU_A_/L and IgE to pre-exposure levels within 1 to 2 years; however, some individuals retained highly elevated levels for many years.

### Booster on Re-exposure

When an IgE-sensitized individual is exposed to an allergen, a booster of the IgE antibody production is expected to take place. However, in sharp contrast to the 2- to 3-fold increase in IgE antibodies to timothy seen after the timothy grass pollen season in timothy allergic individuals, one third of the recommended daily dose of PHO taken during 1 week resulted in a close to 100-fold increase in serum levels of IgE antibodies to PHO and QAI within 3 weeks and equally so of IgE in all of the 13 sensitized patients exposed [9,11]. No effect was seen by codeine, noscapine, and guaifenesin in the 8 IgEsensitized patients or even by PHO in the 2 nonsensitized controls. In addition, serum IgE antibodies to common allergens like house dust mite and cat dander that could not be detected (ie, <0.1 kU_A_/L) before PHO exposure could reach levels in the order of 3 to 5 kU_A_/L after exposure, indicating a polyclonal response.

The mechanisms by which PHO activates IgE synthesis are not known, but the exposure results indicate that the "tail," the morpholino side chain, added to the MOR molecule (Figure 1) to create PHO might be involved. The results further indicate that PHO in an individual IgE sensitized to PHO has a most pronounced effect on the control mechanism for IgE production. As a result, the number of individuals with high levels of IgE antibody to QAI to be at risk for anaphylaxis to NMBA will increase. Similarly, it is theoretically possible that in atopic individuals, the risk for and severity of an IgE-mediated allergic reaction might increase, and equally so the number of blood donors whose blood plasma could passively transfer a temporary allergy to the recipient [12].

The effect on the mechanisms controlling the IgE production was remarkable. Such an IgE booster phenomenon has previously been seen only in the graft-versus-host disease during rejection of a bone marrow transplant [13] but also in angioblastic lymphadenopathy upon cytostatic treatment. The complete clinical importance of this impact is not known, but we strongly suggest that such a serious adverse drug effect is avoided, especially because there are several alternative drugs available OTC for the treatment of mild nonproductive cough.

### Ratio of QAI to Non-QAI Specificities

According to the structure-function relationship described, the ratio of IgE antibodies to PHO with the 2 specificities, the QAI and the non-QAI epitope, respectively, seem to vary considerably from less than 1/10 in blood donors to approximately 1/3 in those with allergy [6].

A diagnostic consequence of this relationship may be that when MOR is used to screen for IgE antibodies to NMBA (ie, the QAI-containing epitope),[14,15] it is possible that the majority of IgE antibodies bind the non-QAI and not the QAI epitope. Indeed, we have shown that before PHO withdrawal, 5% of blood donors were IgE sensitized (≥0.35 kU_A_/L) to MOR but only 0.4% to SUX (ie, the QAI epitope). In individuals with allergy, the prevalences were 10% and 3.4%, respectively [5,6]. However, it is possible that the assay for IgE antibodies to SUX is not sensitive enough [16]. Also, it cannot be excluded that SUX also is presented *in vivo *with a carrier protein resulting in a modified allergenic epitope that stimulates IgE antibodies with an altered specificity not detected by the SUX available for testing *in vitro *like Patent Blue V [17].

## IgE-Mediated Anaphylaxis to NMBA

During the past 3 to 4 decades, anaphylactic reactions during general anesthesia seem gradually to have increased in frequency with reported prevalences in the order of 1/5000 to 1/100,000 [14,15,18]. However, during this period, the main scientific focus and bulk of publications on anesthetic anaphylaxis originate from a mere handful of countries. Some of these have additionally found it necessary to establish standardized national reporting and diagnostic routines and allergoanesthesiological follow-up units to better handle the challenge [16,18-21]. But from the majority of the nations in the world, there have been none or few published reports indicating that in most countries, NMBA-related anaphylaxis is regarded as a clinical problem of limited magnitude. France, Australia, the United Kingdom, and Norway are the countries with high scientific focus on this topic. All of them, except Norway after PHO withdrawal in 2007, are together with a few others figuring on the definite upper end of the INCB list of PHO consumers (Table 2), whereas the large majority of nations remain non-consumers.

In most of the affected countries, but not all, NMBAs are blamed to cause the majority of reactions [22,23]. Although mainly IgE mediated, most events take place without prior exposure to NMBAs, thus strongly indicating that primary IgE sensitization has taken place by exposure to other environmental factors than anesthetic drugs. The less common reactions reported of atracurium might differ in this respect because they seem to occur in multioperated patients without IgE antibodies to QAI [24].

From Sweden and Norway, 2 interventional studies have been published, 1 retrospective and 1 prospective, showing that removing exposure to PHO within few years significantly decreases IgE sensitization and prevalences of reported anaphylactic reactions to NMBAs [6,7].

The key points of the Scandinavian PHO experiences are therefore as follows: First, and with caution because of the small number of individuals studied, Norwegian estimates indicate that if an individual, unknowingly IgE sensitized to the QAI-epitope (e.g. SUX) through PHO exposure, is subjected to the induction of general anesthesia with SUX intravenously, the risk of an IgE-mediated anaphylactic reaction is increased between 200 and 300 times (ie, from 1/5200 as estimated for the general population to 1/20) [16]. Second, if PHO exposure is stopped, first IgE sensitization and then reports of NMBA-related anaphylaxis goes down significantly as reported in studies from Sweden and Norway [6,7]. Furthermore, in Denmark, an unexposed country, anaphylaxis to NMBAs is indeed very rarely diagnosed [21].

## Conclusions

PHO and NMBA share the major allergenic epitope QAI. The direct effects of PHO on IgE synthesis resulting in a remarkable polyclonal increase in IgE are well documented. However, the mode of action and eventual predisposing individual factors are unknown. The morpholino side chain probably plays a role. An increasing body of evidence suggests an association among PHO consumption, IgE sensitization, and NMBA-induced anaphylaxis. When regulatory authorities assess the raised pharmacovigilance concerns about PHO exposure, the primary step, IgE sensitization, needs to be addressed serologically [25]. This represents direct and reliable monitoring. Counting clinical events down the far end of the biological chain of events, like NMBA-related anaphylaxis, is a much more indirect approach because other disease modifying factors may play roles. In addition, it takes time and is hampered with wellaccepted issues like statistics on rare events and validity of spontaneous reporting systems.

## Competing interests

The authors declare that they have no competing interests.
